# Trends in oral cavity, pharyngeal, oesophageal and gastric cancer mortality rates in Spain, 1952–2006: an age-period-cohort analysis

**DOI:** 10.1186/1471-2407-14-254

**Published:** 2014-04-11

**Authors:** Daniel Seoane-Mato, Nuria Aragonés, Eva Ferreras, Javier García-Pérez, Marta Cervantes-Amat, Pablo Fernández-Navarro, Roberto Pastor-Barriuso, Gonzalo López-Abente

**Affiliations:** 1 Galician Agency for Health Technology Assessment, Galician Health Service, Santiago de Compostela, Spain; 2 Cancer and Environmental Epidemiology Area, National Centre for Epidemiology, Carlos III Institute of Health, Madrid, Spain; 3 CIBER in Epidemiology and Public Health (CIBERESP), Madrid, Spain

**Keywords:** Oral and pharyngeal cancer, Oesophageal cancer, Gastric cancer, Mortality, Age-cohort-period analysis, Change-points, Time trends, Spain

## Abstract

**Background:**

Although oral cavity, pharyngeal, oesophageal and gastric cancers share some risk factors, no comparative analysis of mortality rate trends in these illnesses has been undertaken in Spain. This study aimed to evaluate the independent effects of age, death period and birth cohort on the mortality rates of these tumours.

**Methods:**

Specific and age-adjusted mortality rates by tumour and sex were analysed. Age-period-cohort log-linear models were fitted separately for each tumour and sex, and segmented regression models were used to detect changes in period- and cohort-effect curvatures.

**Results:**

Among men, the period-effect curvatures for oral cavity/pharyngeal and oesophageal cancers displayed a mortality trend that rose until 1995 and then declined. Among women, oral cavity/pharyngeal cancer mortality increased throughout the study period whereas oesophageal cancer mortality decreased after 1970. Stomach cancer mortality decreased in both sexes from 1965 onwards. Lastly, the cohort-effect curvature showed a certain degree of similarity for all three tumours in both sexes, which was greater among oral cavity, pharyngeal and oesophageal cancers, with a change point in evidence, after which risk of death increased in cohorts born from the 1910-1920s onwards and decreased among the 1950–1960 cohorts and successive generations. This latter feature was likewise observed for stomach cancer.

**Conclusions:**

While the similarities of the cohort effects in oral cavity/pharyngeal, oesophageal and gastric tumours support the implication of shared risk factors, the more marked changes in cohort-effect curvature for oral cavity/pharyngeal and oesophageal cancer could be due to the greater influence of some risk factors in their aetiology, such as smoking and alcohol consumption. The increase in oral cavity/pharyngeal cancer mortality in women deserves further study.

## Background

Cancers of the upper gastrointestinal (GI) tract are relatively frequent. The upper gastrointestinal tract usually refers to the oral cavity and pharynx, oesophagus and stomach (though some classifications also include the duodenum). In 2008, gastric cancer was estimated to be the fourth most common cancer worldwide and the second leading cause of death in both sexes [1]. Taken together, oral cavity and pharyngeal cancers ranked eighth in number of new cancer cases and deaths, and oesophageal cancer was the ninth leading cancer in terms of cases and the sixth in terms of deaths [1]. All three tumour sites -particularly oesophageal and gastric cancers- continue to register low survival rates [2-4]. In Spain, these tumour sites together accounted for 11% of all cancer-related deaths among men and 8% among women in 2010 [5].

In Europe, upper GI cancer incidence and mortality rates are higher in men than in women, with this difference being more pronounced among cancers arising in the oral cavity, pharynx and oesophagus [6]. They also share some risk factors, though their relative importance depends on the type of upper GI cancer. Whereas the main risk factors for oral cavity and pharyngeal cancer are alcohol consumption, smoking and human papillomaviruses (HPVs), HPV-16 in particular [7,8], the accepted risk factors for oesophageal cancer include alcohol, smoking, obesity and gastro-oesophageal reflux [9], and those for gastric cancer are *Helicobacter pylori* (*H. pylori*) infection, diet and smoking [10].

In most European countries, oral cavity and pharyngeal cancer mortality registered a pronounced increase from 1950 to 1990, a trend that was more marked among men; indeed, these increases are among the greatest recorded for any neoplasia [11,12]. Oral cavity and pharyngeal cancer mortality also increased in countries such as Canada and Australia, though to a lesser extent [13]. The trend has since varied among countries, i.e., while mortality has declined in most Western European countries, it has continued to rise in Central and Eastern Europe [14,15]. Although incidence and mortality trends in oral and pharyngeal cancer have been classically attributed to changes in prevalence of exposure to tobacco and alcohol (the main risk factors for this group of malignancies), some authors have linked recent increases in the incidence of these cancers to HPV infection [16].

In the case of oesophageal cancer, incidence and mortality rates have remained stable in most European Western countries over the last few decades, albeit with some differences [17]. Gastric cancer rates, in contrast, have been steadily declining for the last 50–60 years [18,19], a fact that has been associated with increases in the quality of life in Western countries, including better diet, and the decline in prevalence of *H. pylori* infection [10].

This study used age-period-cohort models to analyse mortality time trends in oral cavity and pharyngeal, oesophageal and stomach cancers in Spain over the period 1952–2006, and compare similarities and differences in the birth cohort and period effects.

## Methods

### Mortality and population data

Population and mortality data for this study are publicly available from the Spanish National Statistics Institute (*Instituto Nacional de Estadística*). During the calendar period considered (1952–2006), three different Revisions of the International Classification of Diseases (ICD) were used. Consequently, the cancer-related deaths studied respectively corresponded to: ICD-6-7 codes 140 to 148, ICD-8-9 codes 140 to 149 and ICD-10 codes C00 to C14 for lip, oral cavity and pharyngeal cancer; ICD-6-9 code 150 and ICD-10 code C15 for oesophageal cancer; and, lastly, ICD-6-9 code 151 and ICD-10 code C16 for stomach cancer. The number of deaths due to selected codes during the study period, broken down by age, gender and calendar period, were obtained from the Spanish National Statistics Institute. Spanish population data corresponding to censuses and municipal rolls for the midyear of each quinquennium were also obtained from the Spanish National Statistics Institute. Mortality and population data were stratified by age group (from 0–4 to 85+ years), sex, calendar period (in eleven 5-year periods, i.e., 1952–1956, 1957–1961,…, 2002–2006) and cancer site. Age-adjusted mortality rates (per 100,000 population, standardised to the European Standard Population) for cancers of the oral cavity and pharynx, oesophagus and stomach were calculated for each sex and 5-year calendar period.

### Age-period-cohort (APC) models

Separate log-linear Poisson models were fitted to study the effect of age, period of death and birth cohort for each sex and tumour site on mortality. Age-specific mortality rates per 100,000 population for the eleven 5-year periods considered were used for the APC analysis. To address the “non-identifiability” problem (i.e., the three factors -age, period and cohort- are linearly dependent), we used Osmond and Gardner’s solution [20], as well as curvature effects and net drift as proposed by Holford [21]. The Osmond-Gardner solution splits net drift into cohort and period slopes, by minimising any disagreement in parameter estimates between the full three-factor model and each of the two-factor models (age-period, age-cohort and period-cohort). Then, it is possible to determine two estimable parameters not affected by the non-identifiability problem: (i) overall change over time (denominated net drift), which is the sum of the cohort and period slopes [21]; and (ii) deviation of any period or cohort estimators from the general trend (denominated curvature). To display the cohort and period effects graphically, we used this solution and the respective curvatures. Age groups <30 years, as well as those <20 years for stomach cancer, were excluded from this analysis due to the limited number of deaths in these age groups. The open-ended category of persons aged 85 years and over was also excluded. We checked for extra-Poisson dispersion [22] and, where present, effects were calculated using a negative binomial distribution.

### Curvature change points

The presence of change points in the curvatures of the cohort and period effects was evaluated by fitting segmented models to the relationship between curvature effect and time. Details of the recursive algorithm used to estimate the segmented regression have been published elsewhere [23], and the procedure can be easily fitted using the R package “segmented” [24]. The models provided: a) the corrected P value of the Davies’ test for the change point; and 2) the estimate and 95% confidence interval for the location of the change point.

## Results

From 1952 to 2006, there were 71,500 deaths in Spain due to cancer of the oral cavity and pharynx, 71,997 due to cancer of oesophagus, and 409,998 due to stomach cancer.

**Figure 1:**
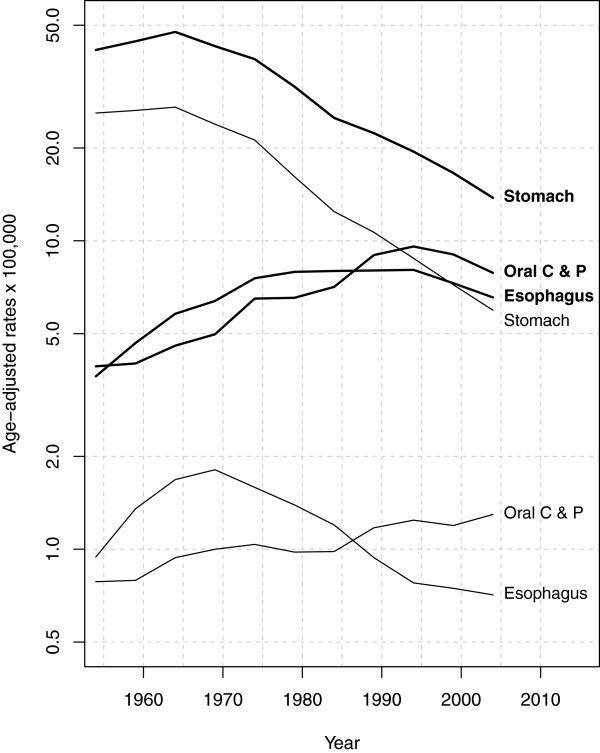
Temporal trends in age-standardised mortality rates per 100,000 person-years (European standard population) for cancers of the oral cavity and pharynx, oesophagus and stomach in males (thick lines) and females (thin lines), Spain 1952–2006.

Table 1 and Figure 1 show the trend in adjusted upper GI cancer mortality rates among men and women. The following aspects should be noted: 1) the higher frequency of all three cancers among men, with the respective male–female ratios for the first and last 5-year periods being 5.0 and 6.0 for oral cavity/pharyngeal cancer, 3.9 and 9.2 for oesophageal cancer, and 1.6 and 2.3 for gastric cancer; 2) the fact that mortality due to oral cavity, pharyngeal and oesophageal cancers rose among men until 1995 and declined thereafter; 3) the increase in oral cavity and pharyngeal cancer rates, which surpassed those for oesophageal cancer in 1986 among men and women; 4) the diverging oesophageal cancer mortality trends in men and women from the 1970s onwards; and 5) the decline in stomach cancer mortality in both sexes since 1965.

**Table 1:** Age-adjusted mortality rates per 100,000 person-years (European standard population) and number of deaths in Spanish men and women, for upper gastrointestinal tract cancer mortality, 1952-2006

	**Men**			**Women**		
	**Oral cavity & pharynx**	**Oesophagus**	**Stomach**	**Oral cavity & pharynx**	**Oesophagus**	**Stomach**
	**Rate**	**Rate**	**Rate**	**Rate**	**Rate**	**Rate**
	**(Deaths)**	**(Deaths)**	**(Deaths)**	**(Deaths)**	**(Deaths)**	**(Deaths)**
**1952-1956**	3.92	3.64	41.56	0.78	0.94	25.96
	(1,697)	(1,609)	(18,514)	(454)	(561)	(15,254)
**1957-1961**	(4.00)	(4.66)	(44.36)	(0.79)	(1.35)	(26.46)
	(1,905)	(2,288)	(21,670)	(506)	(866)	(17,144)
**1962-1966**	(4.57)	(5.80)	(47.56)	(0.94)	(1.68)	(27.12)
	(2,446)	(3,190)	(25,878)	(662)	(1,220)	(19,674)
**1967-1971**	(4.21)	(6.38)	(42.77)	(0.88)	(1.81)	(23.88)
	(2,972)	(3,896)	(25,881)	(785)	(1,462)	(19,371)
**1972-1976**	(5.30)	(7.56)	(38.81)	(0.90)	(1.59)	(21.22)
	(4,334)	(4,991)	(25,205)	(908)	(1,416)	(18,984)
**1977-1981**	(5.87)	(7.93)	(31.63)	(0.90)	(1.39)	(16.15)
	(4,840)	(5,850)	(22,995)	(948)	(1,413)	(16,324)
**1982-1986**	(7.09)	(7.98)	(25.03)	(0.98)	(1.20)	(12.44)
	(5,853)	(6,606)	(20,829)	(1,092)	(1,437)	(14,752)
**1987-1991**	(8.99)	(8.01)	(22.34)	(1.17)	(0.94)	(10.66)
	(7,992)	(7,217)	(20,669)	(1,418)	(1,278)	(14,240)
**1992-1996**	(9.58)	(8.05)	(19.46)	(1.24)	(0.78)	(8.77)
	(9,049)	(7,798)	(19,855)	(1,661)	(1,139)	(13,088)
**1997-2001**	(9.03)	(7.29)	(16.60)	(1.19)	(0.75)	(7.20)
	(9,243)	(7,696)	(18,891)	(1,756)	(1,176)	(11,896)
**2002-2006**	(7.87)	(6.55)	(13.78)	(1.30)	(0.71)	(5.95)
	(8,893)	(7,681)	(17,773)	(2,086)	(1,207)	(11,021)
**Total (Deaths)**	(59,224)	(58,822)	(238,250)	(12,276)	(13,175)	(171,748)

**Figure 2:**
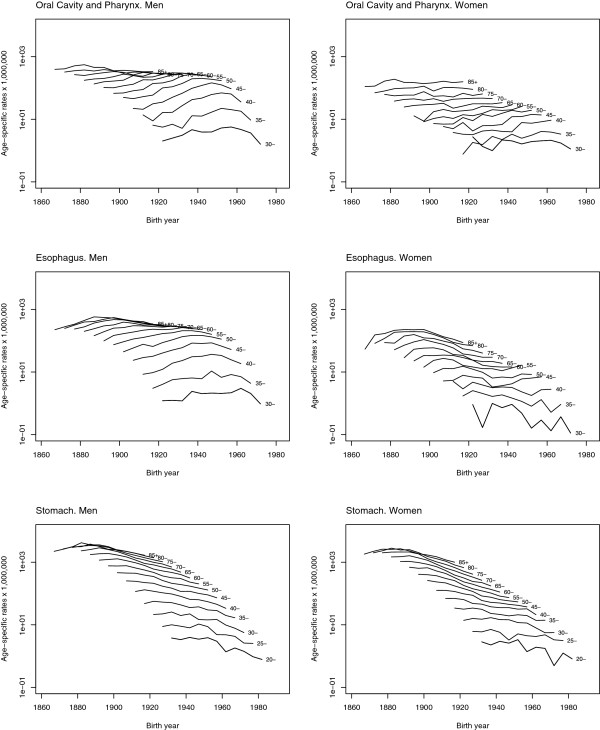
Age specific mortality rates for cancers of the oral cavity and pharynx, oesophagus and stomach by sex, Spain 1952–2006.

The specific mortality rates depicted in Figure 2 enabled trend patterns linked to age and birth cohort effect to be identified. For all three tumours and in both sexes there was an age effect of increasing risk, more markedly until 55 years of age. The graphs corresponding to oral cavity, pharyngeal, and oesophageal cancer were very similar in men and women, and displayed a cohort effect which increased until generations born in the 1950s and then subsequently decreased in men, while in women specific mortality rates increased in the last years of the study. The graphs corresponding to gastric cancer were similar between men and women and showed a more marked decrease among older age groups and in women.

**Figure 3:**
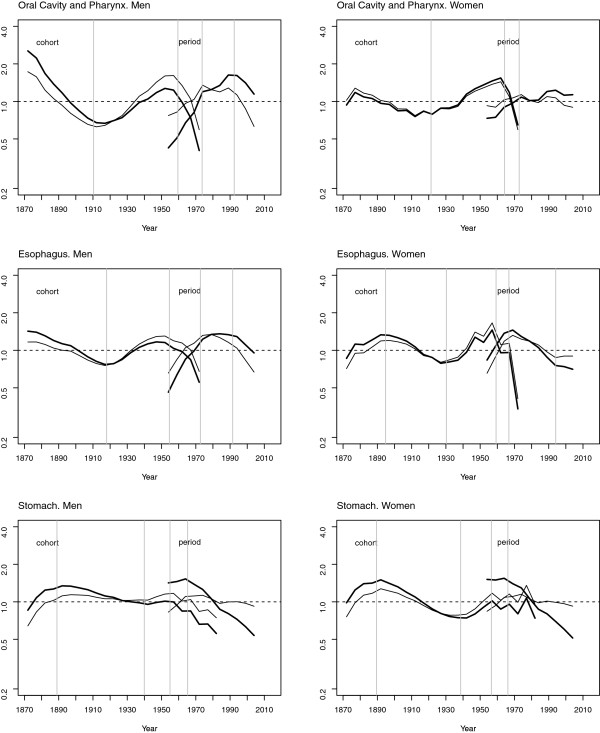
Cohort and period effects (thick lines), curvature (thin line) and change points (vertical grey lines) on cancers of the oral cavity and pharynx, oesophagus and stomach by sex, Spain 1952–2006.

The above patterns can be more easily appreciated in Figure 3, which depicts the results of the age-period-cohort models. This figure shows the cohort and period effects together with their curvatures and change points, and enables the similarity of the shape of the cohort effects in the three tumours to be discerned, though the similarity is greater between oral cavity/pharyngeal and oesophageal cancers in both sexes. A change point can be seen, marking a rise in risk among the 1910–1920 cohorts and a fall in risk among the 1950–1960 cohorts. This latter pattern was also observable for stomach cancer.

Table 2 shows the deviances of the different models used. In all cases, the model that provided the best fit to the data was the age-period-cohort model. The cohort and period effects were statistically significant for all three tumour sites studied.

**Table 2:** Goodness of fit for age-period-cohort models to upper gastrointestinal tract cancer mortality, Spain 1952-2006

**Model**	**Oral cavity & pharynx**		**Oesophagus**		**Stomach**	
**Men**	**D.f.**	**Deviance**	**D.f.**	**Deviance**	**D.f.**	**Deviance**
Age	110	7185.8	110	3052.2	130	19676.3
Age + drift	109	3296.2	109	2396.6	129	1923.0
Age + period	100	2145.2	100	1080.9	120	571.4
Age + cohort	90	2404.7	90	2087.0	108	850.4
Age + period + cohort	81	200.6	81	143.0	99	136.8
Annual net-drift*		*2.01*%		*0.72*%		*-2.70*%
**Women**						
Age	110	492.8	110	1805.0	130	21345.9
Age + drift	109	312.3	109	860.2	129	2048.3
Age + period	100	287.8	100	319.5	120	1077.6
Age + cohort	90	164.6	90	397.8	108	611.1
Age + period + cohort	81	115.1	81	123.6	99	152.4
Annual net-drift*		*1.04*%		*-1.89*%		*-3.50*%

D.f. – Degrees of freedom.

*Overall annual percent change in age-adjusted mortality rates obtained from the sum of period and cohort linear slopes from the three-factor model.

Finally, the trend in the period effect was different for the three tumours and in line with that described above for the adjusted rates. Tables 3 and 4 show the change points detected using segmented regression.

**Table 3:** Cohort effect points of change on upper gastrointestinal tract cancer mortality by sex, Spain 1952–2006

**Tumour**	**Changes in cohort effect†**		
	**Birth year**	**Birth year**	**Birth year**
	**(95% CI)**	**(95% CI)**	**(95% CI)**
Oral cavity & pharynx			
Men	1910.9 (1908.4 - 1913.4)	1956.8 (1955.1 - 1958.5)	
Women	1921.3 (1916.3 - 1926.4)	1964.2 (1962.5 – 1966.0)	
Oesophagus			
Men	1919.2 (1915.3 - 1923.2)	1950.8 (1947.9 - 1953.6)	
Women	1897.3 (1888.5 - 1906.2)	1929.9 (1922.8 - 1937.0)	1959.3 (1956.2 - 1962.3)
Stomach			
Men	1889.0 (1886.1 - 1891.9)	1940.0 (1931.9 – 1948.0)	1954.9 (1950.5 - 1959.4)
Women	1889.5 (1884.7 - 1894.3)	1938.6 (1932.2 - 1945.0)	1956.5 (1946.5 - 1966.6)

**†** Year of birth with significant trend change as obtained from the segmented regression analysis of cohort curvatures from the three-factor model.

**Table 4:** Period effect points of change on upper gastrointestinal tract cancer mortality by sex, Spain 1952–2006

**Tumour**	**Changes in period effect†**	
	**Year of death**	**Year of death**
	**(95% CI)**	**(95% CI)**
Oral cavity & pharynx		
Men	1974.2 (1968.9 - 1979.5)	1994.6 (1990.3 - 1998.9)
Women	1972.7 (1963.2 - 1982.3)	
Oesophagus		
Men	1972.7 (1969.8 - 1975.5)	1991.6 (1987.5 - 1995.6)
Women	1966.8 (1965.6 - 1967.9)	1994.1 (1985.8 - 2002.4)
Stomach		
Men	1965.2 (1963.1 - 1967.4)	
Women	1966.2 (1963.6 - 1968.8)	

**†** Year of death with significant trend change as obtained from the segmented regression analysis of period curvatures from the three-factor model.

## Discussion

The results of this study show the time trend in upper GI tumours in Spain, characterised by: a rise in mortality due to oral cavity, pharyngeal and oesophageal cancer among men until 1995 and its subsequent decline; an increase in oral cavity and pharyngeal cancer rates, which surpassed those of oesophageal cancer in 1986 for men and women alike; an increase in recent decades in oral cavity and pharyngeal cancer among women; diverging oesophageal cancer mortality trends in men and women from the 1970s onwards; and a decline in stomach cancer mortality in both sexes.

The age-period-cohort analysis reported in this study shows that for the period effect there is a certain similarity between the sexes in the tumour sites studied, though there are also some differences. Firstly, there has been no decline in oral cavity and pharyngeal cancer mortality risk among women in recent periods. This divergence between men and women in the trend in oral cavity and pharyngeal cancer may reflect an increase in smoking and alcohol consumption among women, an increase in exposure to HPV, or both.

Secondly, note should be taken of the divergence between men and women in the oesophageal cancer mortality trend as from 1970. Even though it is accepted the influence of smoking and alcohol in the aetiology of this tumour, the incorporation of women to tobacco habit that occurred in Spain during the last decades of the XX century is not reflected in the evolution of oesophageal cancer mortality rates, probably reflecting the lower consumption of alcohol among females [25].

Finally, insofar as the period effect in gastric cancer is concerned, the effects are similar in both sexes, reflecting a lesser implication of lifestyle habits in this tumour and a greater role of the exposure to *H. pylori* in its aetiology. During the last decades, a parallel fall in gastric cancer mortality rates in Spain in high and low risk areas and in both sexes has been observed [26], supporting the possible implication of a constant decrease in *H. pylori* infection rates and a continuous increase in life-standard indicators in the trends of gastric cancer incidence and mortality.

With respect to the cohort effects, attention should be drawn to the similarity of the results for both sexes in all three tumour sites, with the resemblance between the cohort effects in oral cavity/pharyngeal cancer and oesophageal cancer being especially noteworthy, with 2 waves and coincidence of change points. This similarity in the shape of the cohort effect across the three tumour sites suggests exposure to shared risk factors, mainly alcohol consumption and smoking, which are risk factors for oral cavity and pharyngeal cancer, epidermoid carcinomas of the oesophagus [27,28] and, to a lesser extent, gastric cancer [29,30]. Moreover, whereas the change point in the cohort effect in oral cavity, pharyngeal, oesophageal and stomach cancers among men was located in the 1910, 1920 and 1940 generations respectively, among women it was located in generations born around 1920, 1930 and 1940. This correlative change in the trend in younger generations of women might be related to the lag in the increase in the prevalence of smoking and alcohol consumption among women in Spain.

The aim of this study was to compare the trend and period and cohort effects of upper GI tumours. When it comes to interpreting the results, it must be borne in mind that our understanding of the epidemiology of upper GI tract cancers has changed over recent decades. With respect to tumours of oral cavity and pharynx, it is currently accepted that there are two groups of malignant tumours at this site, namely, those associated with smoking and alcohol consumption, and those associated with HPV infection [31]. Cases associated with HPV infection have been reported to be younger and have a better prognosis than HPV-negative cases [32,33]. The decrease in exposure to smoking and alcohol, along with changes in legislation, has resulted in a decline in the incidence and mortality rates of associated SCCs. At the same time as this has been occurring, however, there has been an increase in incidence and mortality in respect of some cancer sites included in the oral cavity and pharyngeal cancer group, due to HPV infection [31]. In Sweden, for instance, a highly significant and parallel increase has been reported in both the incidence of tonsillar and base-of-tongue SCC cancers and the proportion of HPV-positive tumours [34,35]. Furthermore, on studying the trend in HPV-positive tonsillar SCC in the County of Stockholm, Nasman *et al.* found a doubling of HPV-positive cases between 1970 and 2007, accompanied by a parallel decrease in the proportion of HPV-negative tumours [36].

The evidence of a role for HPV in the pathogenesis of oral cavity and pharyngeal cancers is thus both molecular and epidemiological. If the increase in the proportion of HPV-related cases had occurred among younger persons with a better prognosis, these changes would thus account for the second wave of the cohort effect and the subsequent decline in men and women alike. Moreover, if the increase in oropharyngeal cancer mortality in women were found to coincide with an increase in survival, one could speculate that the rise in incidence should be even higher.

HPV, one of the most frequent sexually transmitted infections world-wide [37], is a recognised risk factor for oral cavity and oropharyngeal cancer [38,39]. Although the association between HPV and different cancers is accepted, the epidemiology and natural history of human papillomavirus infection is not yet well understood [40]. Over 100 HPV types have been described, some being highly oncogenic, with a role in the aetiology of anogenital (mainly cervical) [38,41] and oropharyngeal neoplasms [8]. Both genital and oral HPV infection are predominantly sexually transmitted [42]. With regard to oral HPV infection, though infection resolves over time in most cases, HPV infection persists in a small percentage of individuals; and, while it is not known which factors may affect the persistence of the infection, both pathogen and host factors, as well as some environmental co-factors, may possibly be implicated [40].

Regarding knowledge of the prevalence of HPV infection in the general population, a broad range of estimates can be found, depending on study characteristics, geographical area, calendar period, age group, testing technology, etc., since HPV prevalence depends on the cultural, sexual and lifestyle habits of the populations, which also vary over time. World-wide prevalence of genital HPV infection in women with normal cytological findings has been estimated to be around 11.7% [43]. With regard to oral HPV infection, a global prevalence of 4.5% was reported for the general adult population world-wide [44], though this estimate was greater in developing (7.3%) than in developed nations (3.6%). A recent study in the United States reported an overall prevalence of oral HPV infection of 6.9% among men and women aged 14–69 years, with men having a higher prevalence than women (10.1% vs. 3.6%). While the prevalence of genital HPV infection in Spain has been reported to be among the lowest in Europe [45,46], changes in sexual lifestyles in young Spanish cohorts, with higher risk of HPV infection, have nevertheless been reported [47].

Since a clear association between HPV and oropharyngeal SCC has been accepted, the histological similarities between the oral squamous epithelium and upper oesophagus would suggest a similar association [48]. Yet, even though studies that have assessed the role of HPV in oesophageal SCC have, as in the case of oral cavity and pharyngeal cancers, found virus in samples of tumour tissue, the evidence accumulated to date does not allow for a similar association to be inferred [39]. In Spain, whereas incidence of oesophageal SCC in males rose from 1980 until 1986 and then began to decline, among females it increased from 1980–1994 and remained stable from 1994–2003 [49], describing a trend that is more like that of oral cavity and pharyngeal cancer mortality than that of oesophageal cancer as a whole. There may possibly be other types of HPV implicated in oesophageal cancer [50], in much the same way as HPV6 has been associated with laryngeal cancer [39]. Further studies are thus needed to understand the natural history of the virus and its role in oesophageal carcinogenesis.

One specific aspect of the gastric cancer mortality time trend is the change point observed in the cohort effect in generations born from 1940 to 1955, which would correspond to the aftermath of the Spanish Civil War (1936–1939) and would highlight the importance of exposure to risk factors at early ages of life. *H. pylori* infection rates have been reported to be higher during childhood, and they are inversely associated with hygiene practices [51]. In many European Union countries small shifts in the downward trend in gastric cancer mortality have similarly been observed in generations born around the 1940s, which could also be related to worse living conditions in the first years of life among generations born during and immediately after the Second World War [18].

One aspect that also characterises the epidemiology of the three tumour sites studied are the higher incidence and mortality rates among men than among women, with this difference being smaller for gastric cancer [6]. On comparing the last to the first five-year period studied, a rise will be seen in the male–female ratio, particularly in the case of oesophageal cancer. This difference would, in part, be explained by the higher population attributable risk of oral cavity, pharyngeal and oesophageal cancer for smoking and alcohol consumption among men than among women [27,28], though other hypotheses could be considered. Marur *et al.*[31] suggested that higher HPV prevalence in cervical as opposed to penile tissue might boost the chances of HPV infection in men when performing oral sex, and so contribute to the higher rate of HPV-related oropharyngeal cancer in men.

In order to show the results of the age-period-cohort models, we opted for Osmond and Gardner’s solution, as well as the evaluation of estimable parameters proposed by Holford. The existence of different solutions is a source of uncertainty, since all of them are subject to limitations and it is difficult to ascertain which is the most appropriate. Even so, there are many components of the information depicted graphically that do not vary across the different solutions, e.g., the shape of the cohort effect (local changes or curvature) is independent of the solution chosen. Hence, where a trend is observed in the cohort effect, generally speaking this remains relatively unchanged in the different solutions. The graph-based representation of the cohort effect makes it possible to detect the generation marking the shift in trend. The same applies to period effects -change or inflection points in the trend- which remain visible throughout.

When it comes to interpreting the extreme values of the cohort effects (the oldest and youngest cohorts) in this type of analysis, one must bear in mind that these are estimated with a single cell of the rates matrix and so are extremely unstable. This is especially so in the case of the extreme values of the youngest cohort, since these are calculated on the basis of very few cases. Consequently, any sharp changes at the extremes of the cohort effects must be interpreted with great caution, particularly those limited to the latest birth cohorts (e.g., the youngest cohorts in women).

One strength of this study is that it involves the follow-up of the total Spanish population across 55 years. This is thus a dynamic cohort, with entries and exits across the study period, which encompasses generations born approximately from 1865 to 1985 and thus constitutes an important time series. At the same time, the fact that its spans such a long time period means that some of the results might be due to changes in the quality of death registration, in the coding of causes of death, and/or in survival. At all events, mortality data are the only data that enable such a long series to be studied in the Spanish population as a whole, and the quality of such data for the case of cancer has not only been demonstrated, but has also been shown to be similar to that of other countries in the region [52].

Furthermore, the low survival rate means that mortality due to the causes studied is a good approximation of incidence. In the period 1995–1999, relative age-adjusted survival at 5 years in Spain was 9.7% for oesophageal cancer and 27.8% for stomach cancer. For oral cavity and pharyngeal cancer, survival was higher (36.5% in men and 53.1% in women) [2,3], which could explain the change in the oral cavity and pharyngeal age adjusted mortality rates trend among men and women from 1995 onwards.

## Conclusion

In conclusion, the similarities displayed by the trends in the tumours studied -especially those of the oral cavity, pharynx and oesophagus- would support the implication of shared risk factors. Many of the risk factors described are preventable factors, such as smoking and alcohol consumption. Furthermore, HPV vaccination was approved in Spain in 2007 for the prevention of cervical cancer [53]. Though the protective role of HPV vaccination against nongenital cancers is still under study, it might have an effect on the prevention of all HPV-associated malignancies. However, such HPV vaccination programmes target women. In future, monitoring should focus on trends in non-cervical HPV-related neoplasms, in both men and women, particularly at cancer sites with rising incidence rates. From a health-promotion standpoint, it might also be advisable to educate the general population about HPV transmission routes and the role of HPV in the aetiology of oral cavity and pharyngeal cancer, as well as dentists and physicians who diagnose and treat head and neck cancers, since effective screening modalities are limited in some of these sites.

Our results highlight the importance of continuing to study the causes implicated in the aetiology of these tumours, as well as the need to insist on primary prevention measures targeting known risk factors and secondary prevention measures.

## Competing interests

The authors declare that they have no competing interests.

## Authors’ contributions

GLA and NA designed the study. GLA, DSM, JGP and PFN performed the statistical analysis. DSM and NA wrote the first draft of the manuscript to which all authors subsequently contributed. All authors made contribution to statistical analyses and interpretation of results, and revised the manuscript for important intellectual content. All authors read and approved the final manuscript.

## Pre-publication history

The pre-publication history for this paper can be accessed here:

http://www.biomedcentral.com/1471-2407/14/254/prepub
